# The pathogenicity of blood stream and central nervous system forms of *Trypanosoma brucei rhodesiense* trypanosomes in laboratory mice: a comparative study

**DOI:** 10.12688/f1000research.75518.1

**Published:** 2022-03-02

**Authors:** Kariuki Ndungu, John Thuita, Grace Murilla, John Kagira, Joanna Auma, Paul Mireji, Geoffrey Ngae, Paul Okumu, Purity Gitonga, Samuel Guya, Raymond Mdachi

**Affiliations:** 1 Biochemistry, Kenya Agricultural and Livestock Research Organization, Nairobi, P.O. Box 362 -00902, Kenya; 2 Animal Science, Meru University of Science and Technology, Meru, P.O Box, 972-60200, Kenya; 3 Administration, KAG East University, Nairobi, P.O.BOX 46328-00100, Kenya; 4 Animal Science, Jomo Keyatta University of Science and Technology, Nairobi, P.O. Box 62000–00200, Kenya; 5 Bioinformatics, Centre for Geographic Medicine Research, Kilifi, P. O. Box 428-80108, Kenya; 6 Food Crops Research Institute, Kenya Agricultural and Livestock Research Organization, Nairobi, P. O. Box 30148-00200, Kenya; 7 Veterinary Pathology, University of Nairobi, Nairobi, P.O. Box 30197-00100, Kenya

**Keywords:** Trypanosoma, rhodesiense, BSF, CNS, Morphology, pathogenicity.

## Abstract

**Background:** Human African trypanosomiasis (HAT) develops in two stages namely early stage when trypanosomes are found in the blood and late stage when trypanosomes are found in the central nervous system (CNS). The two environments are different with CNS environment reported as being hostile to the trypanosomes than the blood environment. The clinical symptoms manifested by the disease in the two environments are different. Information on whether blood stream are pathologically different from CNS trypanosomes is lacking. This study undertook to compare the inter-isolate pathological differences caused by bloodstream forms (BSF) and central nervous system (CNS) of five *Trypanosoma brucei rhodesiense* ( *Tbr*) isolates in Swiss white mice.

**Methods:** Donor mice infected with each of the five isolates were euthanized at 21 days post infection (DPI) for recovery of BSF trypanosomes in heart blood and CNS trypanosomes in brain supernatants. Groups of Swiss white mice (n = 10) were then infected with BSF or CNS forms of each isolate and monitored for parasitaemia, packed cell volume (PCV), body weight, survivorship, trypanosome length, gross and histopathology characteristics.

**Results:** Amplification of SRA gene prior to trypanosome morphology and pathogenicity studies confirmed all isolates as *T. b. rhodesiense.* At 21 DPI, CNS trypanosomes were predominantly long slender (LS) while BSF were a mixture of short stumpy and intermediate forms. The density of BSF trypanosomes was on average 2-3 log-scales greater than that of CNS trypanosomes with isolate KETRI 2656 having the highest CNS trypanosome density.

**Conclusions:** The pathogenicity study revealed clear differences in the virulence/pathogenicity of the five (5) isolates but no distinct and consistent differences between CNS and BSF forms of the same isolate. We also identified KETRI 2656 as a suitable isolate for acute menigo- encephalitic studies.

## Introduction

Human African Trypanosomiasis (HAT), sleeping sickness) is caused by *Trypanosoma brucei rhodesiense (T.b. rhodesiense, Tbr)* and *T. b. gambiense (Tbg)* species of trypanosomes which are transmitted by tsetse flies ( *Glossina* species). ^1^ *Trypanosoma b. rhodesiense* is predominant in eastern and southern Africa and causes the acute form of HAT while *Tbg* is predominant in Central and Western Africa and is responsible for the chronic form. ^2^ Two stages are recognized in the clinical presentation of HAT, including the hemo-lymphatic (early, stage 1) and the meningoencephalitic (late, CNS, stage 2); the trypanosomes recovered from the haemolymphatic body compartment are typically identified as blood Stream form (BSF) while those recovered from the central nervous system are identified as CNS forms. The early stage infection is clinically non-specific, manifesting as malaise, headache, arthralgia, generalized weakness, weight loss and anaemia. ^3^ On the other hand, late stage infection, occasioned by the parasites crossing the blood-brain barrier or blood- cerebrospinal fluid (CSF) barrier and invading the CNS ^4^^,^^5^ clinically manifest as psychiatric, motor, sensory and sleep abnormalities. ^6^ The CNS invasion may be aided by parasite and/or host derived factors. ^5^

In the CNS, the parasites DNA can be detected as early as between six and seven days post infection (DPI) with parasites being in their replicative slender forms and reaching peak infection at 21 DPI. ^7^ The invasion of the CNS by trypanosomes precipitates changes in the cerebrospinal fluid characterized by presence of cytotoxic compounds, reduced cerebrospinal fluid volume as well as reduced CSF glucose levels ^8^, thus making the CSF more 'hostile' to the trypanosomes. ^8^^,^^9^ In a review by ^10^ it was reported that *invitro*, trypanosomes can only survive for 20hrs in CSF and that this unfavorable nature of CSF could be the cause of parasite migration from the sub-arachnoid space into the pial cell layer. ^11^ The combined effect of these CSF changes on the phenotypic and morphologic characteristics of CNS derived trypanosome forms are poorly understood. In this study therefore, as part of ongoing efforts to characterize the biospecimens at the Kenya Agricultural and Livestock Research Organization -Biotechnology Research Institute (KALRO-BIORI) trypanosome cryobank, we characterized five randomly selected isolates focusing on morphologic and phenotypic changes associated with BSF or CNS trypanosome forms.

## Methods

### Ethics

Approval for performing our experiments on mice was obtained from the Kenya Agricultural and Livestock Research Organization -Biotechnology Research Institute - (KALRO- BioRI) Review Board (C/Biori/4/325/II/49).

### Selection of Trypanosomes isolates

The study used five (5) isolates including *T b rhodesiense* KETRI 3738, KETRI 3537, KETRI 2656, KETRI 3459 and EATRO 2291 ( Table 1). These isolates were randomly selected from the KALRO- BioRI (formerly KETRI) cryo-bank and were originally isolated from HAT patients in the three east African countries of Kenya, Uganda and Tanzania as previously reported. ^12^ The isolates had undergone a minimal 1-8 eight passages in mice ( Table 1).

**Table 1.** Isolates of *T.b. rhodesiense* collected from different parts of eastern Africa.

Stabilate No:	Locality	Year of isolation	Host of isolation	Passage No
**KETRI 3738 (2537)**	Banda, Busoga, Uganda	1972	Human	8
**KETRI 3537**	Bungoma, Western Province, Kenya	1998	Human	3
**KETRI 2656**	Lambwe valley, Kenya	1983	Human	2
**KETRI 3459**	Kitanga, Tanzania	1960	Human	3
**EATRO 2291**	Busoga, Uganda	1976	Human	1

### Molecular characterization of Trypanosome isolates

We validated the isolates *Tbr* species status using PCR as previously described. ^13^ DNA was prepared using QIAGEN DNAesy Blood & Tissue Extraction kit® Cat No 69504. Applied Biosystems Model 2720 thermocyclers was used and the reagents (Pcr Buffer, dNTPs, Mgcl _2_, a pair of primers (SRA A & E),Taq and pcr grade water were from Promega USA. The cycling conditions and reagent concentrations were according to Gibson et al 2002 except the reaction volumes were 10 microlitres per sample. We included DNA from a reference *Tbr* as a positive control KETRI 3738 ^14^ whereas PCR water and *Trypanosoma brucei brucei* ( *Tbb*) were used as negative control. We resolved the amplicons on 2% molecular grade Top vision agarose (Thermo Scientific) stained with ethidium bromide, and documented the gel using UVITEC (Cambridge) gel imager.

### Experimental animals

We obtained 145 male Swiss White mice weighing 25-30g and seven weeks old from KALRO-BioRI Swiss White mice colony which is inbred. The inclusion of an animal was based on the body weight and Packed Cell Volume (PCV) over a period of 14 days of acclimatization. The body weight was in the range of 20-35g and PCV in the range of 45-60%. Animals below 20g bodyweight or 45% PCV were excluded from the experiment. Experimental animals were randomly picked from a pool of mice that fulfilled the inclusion criteria. The personnel taking care of the experimental animals, technicians taking samples and the biometrician who did the analysis were not involved in the proposal development and were therefore unaware of the study objectives. They were housed in standard mouse cages using woodcarvings as bedding materials. The mice were maintained on a diet consisting of commercial pellets (Unga® Kenya Ltd) and water provided *ad libitum.* They were kept in a locked room under natural light. Room temperature and humidity were not regulated. We acclimatized the mice to experimental room conditions for two weeks during which period they were dewormed using ivermectin (Noromectin®, Norbrook, UK) at 0.2mg/kg as previously described. ^15^ At the end of the two weeks acclimatization period, baseline data on packed cell volume (PCV) and body weight were collected.

### Experimental design

Experimental animals were randomly picked from a pool of mice that fulfilled the inclusion criteria (above) and placed in cages containing 10 mice per cage for experimental groups and five (5) mice per cage for the control groups of mice. Donor mice were immunosuppressed using cyclophosphamide injected intraperitoneally at 300 mg/kg for three days consecutively as previously described. ^16^ The immunosuppressed mice were intraperitoneally injected with thawed *Tbr rhodesiense* isolates obtained from the KALRO BioRI cryobank ( Table 1) using four mice per *Tbr* isolate. Donor mice parasitaemia was monitored for 21 days post infection to ensure development of late stage disease as previously described, ^17^ after which the donor mice were placed in a chamber and euthanized using concentrated CO _2_ asphyxiation and in accordance with guidelines of the Institutional Animal Care and Use Committee (IUCAC) and as described by ^18^ euthanized using concentrated carbon dioxide (CO _2_). We collected heart blood, containing the bloodstream form (BSF) trypanosomes in vials containing 5μl of 10% EDTA/mL of blood; blood from the four donor mice injected with a single *T b rhodesiense* strain was pooled into one vial. Blood smears were made from heart blood for morphology studies of BSF trypanosomes.

Intact brains from the four donor mice were also harvested and separately suspended in cold PSG pH 8.0. We then washed each mouse brain tissues for at least ten times in PSG pH 8.0 buffer and microscopically examined each wash for the presence of trypanosomes. When no trypanosomes could be detected, the final buffer wash was discarded, the brain excised using sharp pair of scissors and gently homogenized in PSG pH 8.0. The brain tissue supernatant from the four (4) donor mice were pooled into one vial and used to make smears for morphology studies of CNS trypanosomes.

Trypanosomes density in the pooled heart blood (BSF trypanosomes) and pooled brain supernatant (CNS trypanosome forms) of each *T b rhodesiense* isolate were then quantified using a haemocytometer ( Table 2). The density of BSF trypanosomes was then adjusted using PSG pH 8.0 so that the BSF trypanosome density was equal to that of the CNS trypanosome forms in the brain supernatant. The BSF or CNS trypanosome containing fluids were then used to infect 10 experimental mice per isolate. The mice were inoculated intraperitoneally at 0.2 mLs per mouse. Five non-infected mice were used as controls for the study.

**Table 2.** Trypanosomes density in pooled heart blood (BSF) and central nervous system (CNS).

Isolate	BSF	CNS	BSF/CNS
KETRI 3738	8.5 10 ^7^	1.5 x 10 ^5^	567 (2.75)
KETRI 3537	4.0 x 10 ^7^	1.0 x 10 ^5^	400 (2.6)
KETRI 2656	5.1 x 10 ^8^	7.0x10 ^6^	73 (1.86)
KETRI 3459	8.0 x 10 ^8^	4.0 x 10 ^5^	2000 (3.3)
EATRO 2291	7.0 x 10 ^7^	1.0 x10 ^5^	700 (2.85)

The infected mice were monitored for pre-patent period (PP), parasitaemia progression and survival daily while packed cell volume (PCV) and body weight were measured once in a week. Gross pathology and histopathology were performed at the end of experiment. The control mice were monitored similarly to the infected mice, except for parasitaemia and pre-patent period. At 30 days post infection, we sacrificed 4/10 mice from the infected mice groups and 2/5 mice from the non-infected control group for gross pathology (lesions and organ weights) and histopathology. Such mice were placed in a chamber and euthanized by CO2 asphyxiation and in accordance with guidelines of the Institutional Animal Care and Use Committee (IUCAC).

### Pre-patent period (PP), parasitaemia progression and survival time determination

Blood for estimation of parasitaemia levels was collected daily from each mouse using the tail tip amputation method. ^19^ The PP and parasitaemia levels were determined using the rapid matching method. ^20^ The infected mice were monitored for a maximum of 30 DPI. In our effort to ameliorate any suffering of animals, mice which attained the at extremis end point earlier than this time were sacrificed immediately by CO _2_ asphyxiation in accordance with guidelines of the Institutional Animal Care and Use Committee (IUCAC) as described by ^21^ and recorded as dead animals. The mice were determined to have attained the end point by observation of clinical signs such as lethargy and hackle hair, as well as PCV drop of approximately 25% with consistent high parasitaemia levels of 1 × 10 ^9^/mL for at least three consecutive days. For the survival analysis, mice were monitored at least once per day. ^22^ Mice surviving until the end of the monitoring period of 30 DPI were euthanized using CO _2_ and the survival time categorized as censored data.

### Determination of trypanosomes length

We measured the length of bloodstream form (BSF) trypanosomes recovered from the peripheral blood of mice that were initially infected with BSF or CNS forms of KETRI 3738, KETRI 3537 and KETRI 2656. Thin blood smears were prepared from tail-snip blood and examined using Leica DM500 microscope at high magnification with oil immersion objective (10x100). The length of the trypanosome was measured from the posterior end to the anterior end including the free flagellum as previously outlined. ^23^ On average, 50 trypanosomes of each experimental group of mice were measured.

### Packed cell volume (PCV) and body weight changes

Packed cell volume and body weight changes were determined using a microhaematocrit centrifuge and a weighing balance (Mettler Toledo PB 302 ®, Switzerland) respectively. To ameliorate any suffering of animals,blood sample for the determination of the PCV were collected at a frequency of once a week as outlined previously. ^24^^,^^25^

### Gross and Histopathology

A total of 4/10 mice in each infected mice group and 2/5 of the control mice group were sacrificed at extremis for gross and histopathological examination. The carcass weight of each mouse was determined after which the mouse was dissected and the brain, spleen, kidneys, liver, lungs and heart collected and weighed using a weighing balance (Mettler Toledo PB 302 ®, Switzerland). Carcass and organ weight data and gross pathology lesions were recorded. The organs were preserved in 10% formalin and thereafter processed and examined for histopathology changes as previously described. ^26^ All the tissue lesions observed in the histopathology slides were also recorded.

### Statistical analysis

The data were summarized as means ± standard error of mean, while time bound changes of each of the isolates’ biomarkers of pathogenicity as well as the differences between BSF and CNS trypanosome forms were analyzed using one-way ANOVA. All analyses were conducted using GenStat, Version 15.3 developed by VSN International LTD and licensed to CGIAR, UK where p⩽0.05 were considered statistically significant. R Statistical Software would be alternative free-to-use software. General Linear Model in SAS Release 8.02 was used to analyze data on the length of the trypanosome. Differences between any two means were considered significant at p < 0.05. Survival data analysis was carried out employing the Kaplan–Meier method on StatView (SAS Institute, Version 5.0.1) statistical package for determination of survival distribution function. IBM SPSS would be a good open access software to use. Rank tests of homogeneity were used to determine the effect on host survival time of BSF- and CNS-infected mice. ^27^

## Results

### Molecular identification of cryo-bank isolates

**Figure 1.**
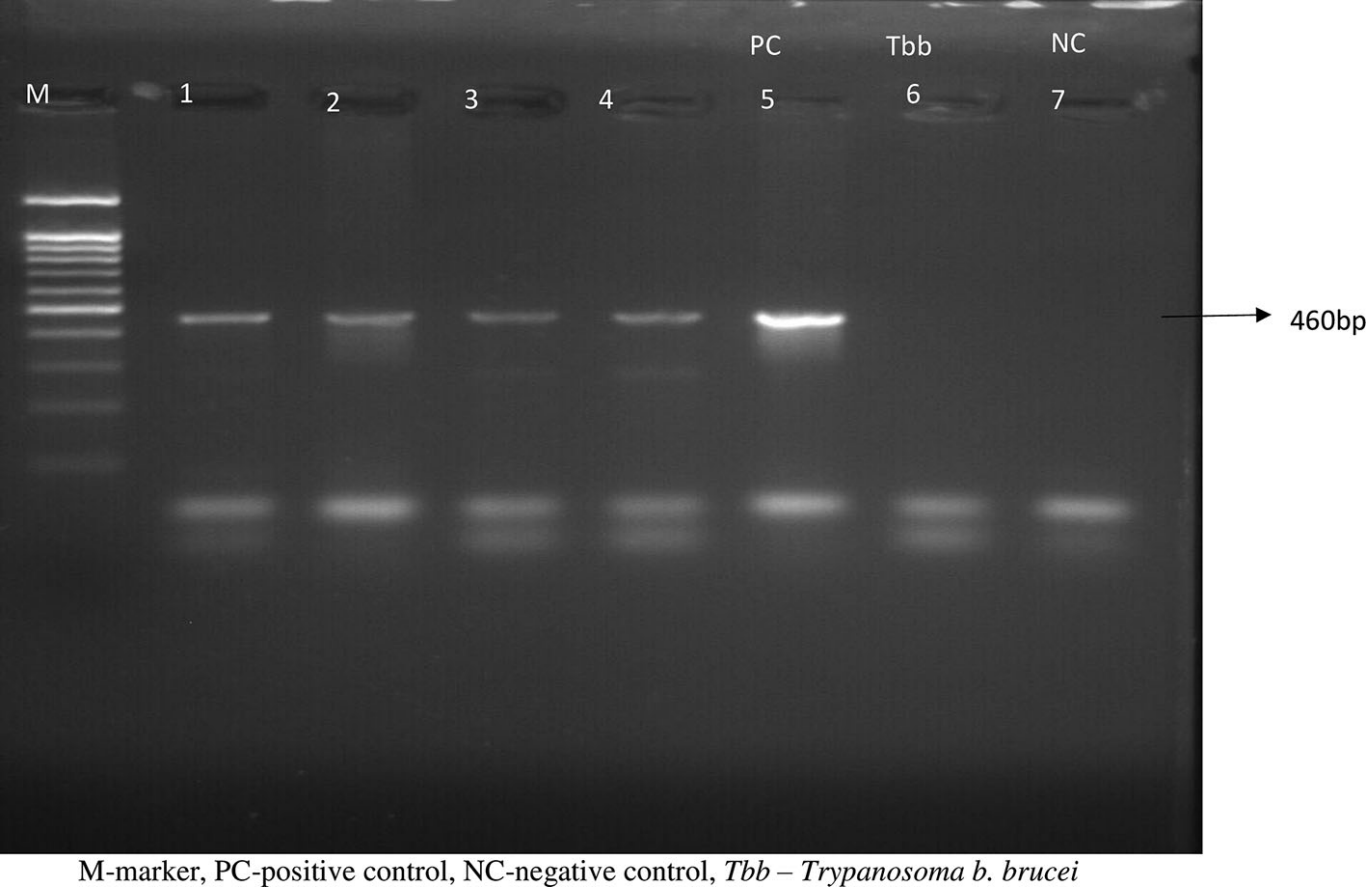
Showing the PCR reactions of the Tbr isolates: M=marker; 1=KETRI 3537; 2=KETRI 2656; 3=KETRI 3459; 4=EATRO 2291; 5=KETRI 3738; PC=Positive control; 6= *Trypanosoma brucei brucei*-Negative control (NC); 7=PCR water.

The 460bp SRA gene fragment was amplified in all the isolates ( Figure 1), confirming them to be *T. b. rhodesiense* isolates. This finding is consistent with KALRO-BioRI cryobank records showing that these isolates were recovered from sleeping sickness patients in eastern African Countries that are endemic for Rhodesian sleeping sickness and it contributes to the continuous efforts to ensure that all the bio specimens in the laboratory are well characterized. ^14^

### Parasite Morphology and density

**Figure 2.**
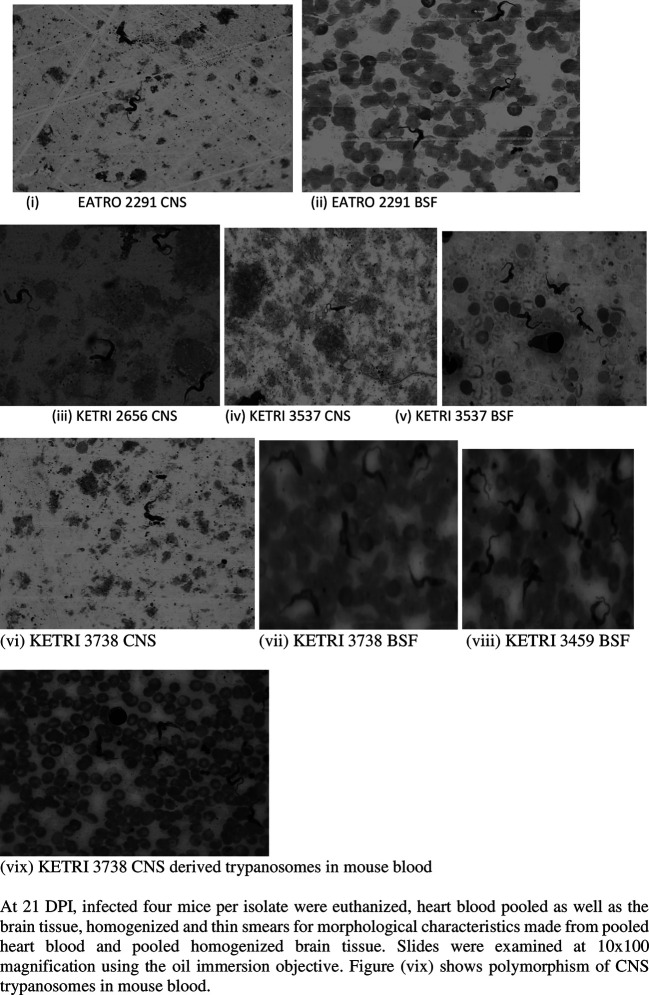
Showing the morphology of BSF or CNS trypanosomes at 21 DPI examined from giemsa stained smears at 10x100 magnification using the oil immersion objective. Abbreviations: BSF, blood stream forms; CNS, central nervous system.

At 21 DPI of the donor mice, the giemsa stained CNS trypanosomes were predominantly long slender while BSF trypanosomes were a mixture of short stumpy and intermediate forms ( Figure 2). With respect to trypanosome density, there were 2-3 times more trypanosomes per field in slide smears of heart blood (BSF forms) as compared to slide smears of brain supernatants (CNS forms) made from the same isolate ( Figure 2). Actual enumeration of trypanosomes using the haemocytometer technique confirmed that the density of trypanosomes in pooled heart blood was 2-3 log scales greater than that of trypanosomes in pooled brain supernatant ( Table 2). When trypanosome density of all the isolates were compared ( Table 2, Figure 2 (iii)), brain supernatants of isolate KETRI 2656 (CNS forms) had a density of 7.0 × 10 ^6^ trypanosomes/mL which was at least 10 times greater than any other isolate ( Table 2). In heart blood (BSF forms), isolates KETRI 2656 and KETRI 3459 had the highest trypanosome densities ( Table 2).

### Pre-patent period of T b rhodesiense isolates in mice

The overall mean ± SE pre-patent period (PP), was 5.2 ± 0.3 and 4.7±0.2 for all the mice that were infected with the BSF or CNS-derived trypanosomes, respectively (p < 0.05).

**Figure 3.**
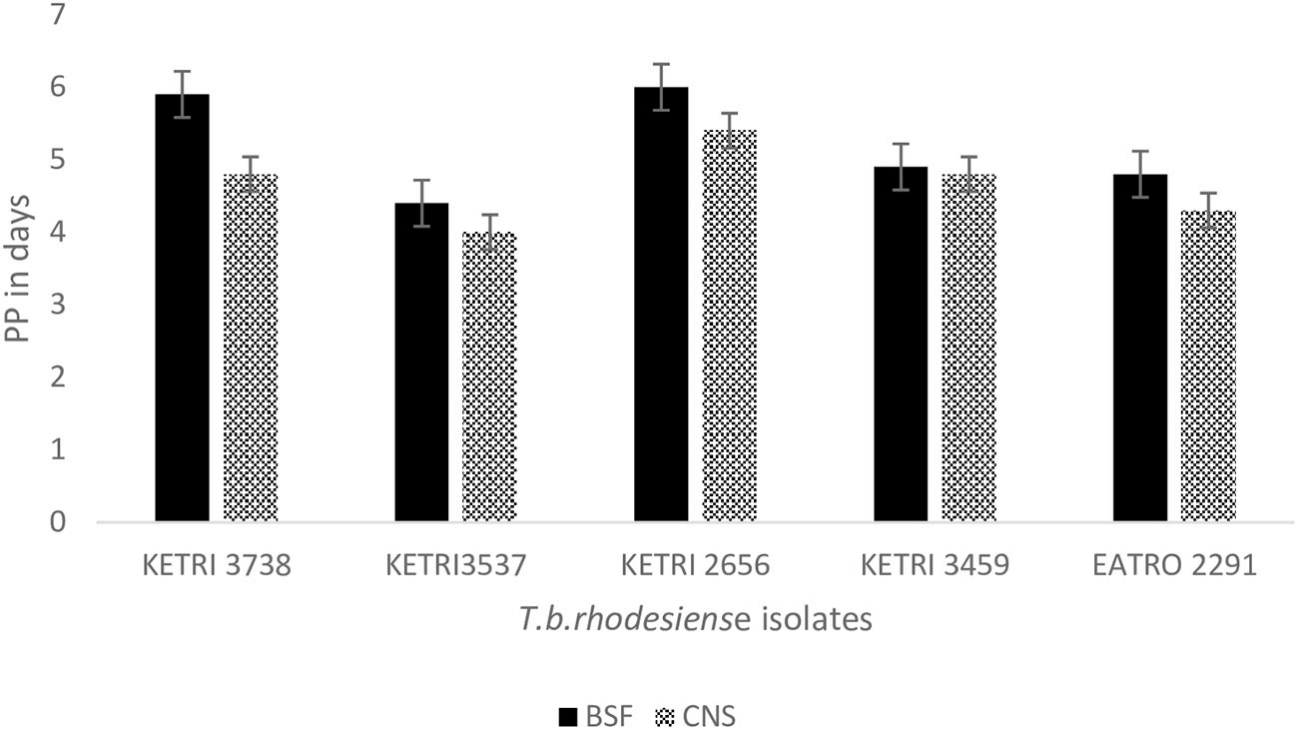
Graph showing the pre-patent period in mice infected with *T. b. rhodesiense* BSF or CNS forms

However, the isolate specific pre-patent period data showed that in 2/5 isolates, KETRI 3738 and KETRI 2656, the PP in mice infected with CNS forms was significantly shorter than the PP in mice groups infected with the BSF forms ( Figure 3). The PP of the remaining 3/5 isolates, KETRI 3537, KETRI 3459 and EATRO 2291, did not exhibit any significant differences (p> 0.05) between BSF and CNS trypanosome forms ( Figure 3). In general, the isolates KETRI 3738 and KETRI 2656 BSF forms had longer PP times compared to the other three isolates, KETRI 3537, KETRI 3459, and EATRO 2291 (p <0.01) ( Figure 3).

### Parasitaemia progression

**Figure 4.**
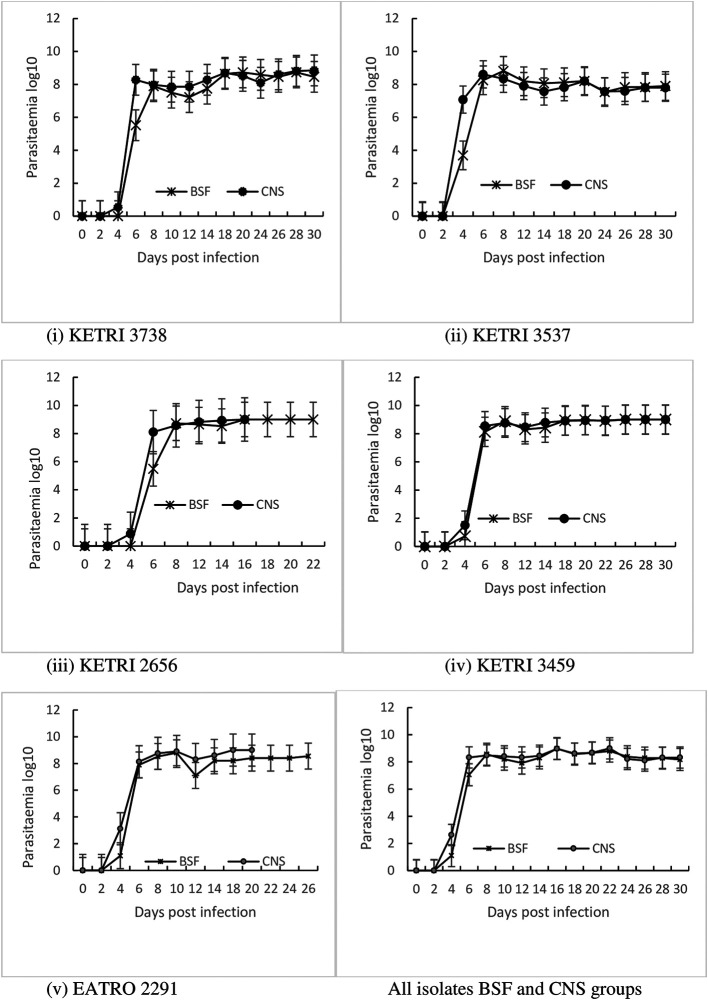
Graph showing the parasitaemia progression in mice infected with *T. b. rhodesiense* BSF or CNS derived trypanosomes. Abbreviations: BSF, blood stream forms; CNS, central nervous system.

Parasitaemia increased rapidly attaining an average first peak parasitaemia of 6.1x10 ^8^ and 6.8x10 ^8^ trypanosomes/mL of blood for both BSF or CNS derived trypanosomes ( Table 3), showing that in general, the parasitaemia patterns were similar. In BSF infected mice, the peak parasitaemia varied between the isolates with the lowest peak (1.6x10 ^8^/mL) recorded in KETRI 3738 infected mice and the highest peak (1.0x10 ^9^/Ml) in EATRO 2291 infected mice. Similarly, in CNS infected mice, the lowest parasitaemia peak (5.0x10 ^8^/Ml) was recorded in KETRI 3738 and 3537 infected mice whereas the highest (1.0x10 ^9^/Ml) was recorded in KETRI 2656 infected mice ( Table 3). On average, the first peak parasitaemia was attained after an average of 7 and 8 days for CNS or BSF trypanosome forms respectively ( Table 3), showing that at the initial stages of the infections, the parasitaemia increase in mice infected with CNS derived trypanosomes was significantly (p < 0.05) faster than those infected with BSF trypanosomes ( Figure 4).

**Table 3.** Parasitaemia and survival time data for mice groups infected with bloodstream and central nervous system forms of *T b rhodesiense.*

Isolate	Time to peak parasitaemia (days)		Mean ± SE of Log _10_ Peak Parasitaemia (Number of trypanosomes/mL of blood)		Mean ± SE Survival time	
					in days	
	BSF form	CNS forms	BSF forms	CNS forms	BSF forms	CNS forms
**KETRI 3738**	8	7	8.2±0.4 (1.6x10 ^8^)	8.7±0.1 (5.0x10 ^8^)	30±0	30±0
**KETRI3537**	7	6	8.9±0.1 (7.9x10 ^8^)	8.7±0.1 (5.0x10 ^8^)	24.6±2.2	26±3.7
**KETRI 2656**	9	9	8.5±0.2 (3.2x10 ^8^)	9±0.03 (1.0x10 ^9^)	18.7±1.4	15.8±0.5
**KETRI 3549**	8	8	8.9±0.1 (7.9x10 ^8^)	8.8±0.04 (6.3x10 ^8^)	28±0.8	26.8±1.0
**EATRO 2291**	9	8	9.0±0.1 (1.0x10 ^9^)	8.9±0.05 (7.9x10 ^8^)	13.5±2.7	11.2±1.4
**Mean ± SE**	**8±0.28**	**7.4±0.39**	**8.76±0.14(6.1x10** ^**8**^ **)**	**8.83±0.06(6.8x10** ^**8**^)	**23±3.0**	**22±3.6**

Number in parenthesis represents the actual parasitaemia score. Abbreviations: BSF, blood stream forms; CNS, central nervous system.

### Trypanosome length

The results of measurement of the length of trypanosomes recovered from the peripheral blood of experimental mice are shown in ( Table 4). The average length of the trypanosomes was a mean ±SE of 26.3±0.23 and 27.5±0.21 for the mice that were initially infected with BSF or CNS trypanosome forms, respectively ( Table 4); these numbers were however not significantly different (p> 0.05). The mean± SEM length of *T. b. rhodesiense* KETRI 3738 trypanosomes was 24.4±0.4 and 25.4±0.3 for mice initially infected with BSF or CNS forms respectively (p> 0.05). Similarly, the mean length of the other isolates KETRI 3537 and KETRI 2656 did not exhibit significant differences between BSF or CNS forms (p> 0.05)

**Table 4.** Trypanosome length by isolate and form.

Isolate	Form		Isolate mean
	BSF	CNS	
KETRI 3738	24.4	25.4	25.0
KETRI 3537	25.4	27.1	26.3
KETRI 2656	29.0	30.0	29.5
Form mean	26.3	27.5	26.9

Abbreviations: BSF, blood stream forms; CNS, central nervous system.

### Packed cell volume (PCV)

**Figure 5.**
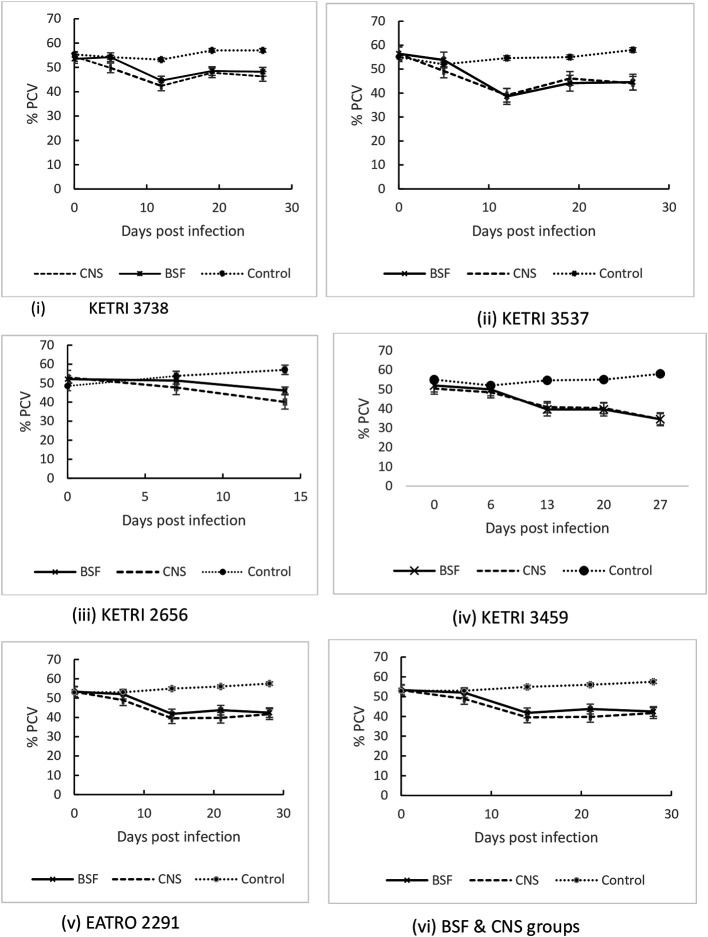
Graph showing the PCV changes in mice infected with BSF or CNS derived trypanosomes with days post infection. Abbreviations: BSF, blood stream forms; CNS, central nervous system. Data are mean ±SE of mice body weight changes (n=10).

The Mean ±SE pre-infection PCV data were 53.2±0.8% and 53.3±1.0 % for BSF or CNS groups respectively; these data were not significantly different (p> 0.05) to Mean ±SE PCV values of 52.9 ± 2.2% for the control group. All mice groups infected with CNS or BSF forms of each *T b rhodesiense* isolates recorded a significant (p < 0.001) decline in PCV within the first 14 days post infection when compared with their pre-infection data ( Figure 5). At 14 DPI, the PCV decline in CNS infected mice ranged from 40.8 ± 1.6 (19%) for KETRI 3459 to 35.3 ± 0.5 (33%) for KETRI 2291. Similarly, the decline in BSF infected mice ranged from 46.1±1.1 (12%) for KETRI 2656 to 38.6±1.4 (32%) for KETRI 3537 Figure 5, ii. After 14 days post infection, the trend of decline and or recovery of PCV was isolate dependent. Overall, the mean (± SE) PCV of CNS infected mice declined from 53.3±1.0 at day 0 to 39.5±1.2 at 14 DPI, which was a 26.9% decline. In the same period, the mean PCV of BSF infected mice declined from 53.2±0.8 to 41.8±1.5 which was a 21.6%. The rate of decline of PCV was therefore significantly (p<0.001) greater for mice that were infected with CNS forms than for mice infected with BSF forms ( Figure 5(vi)). In contrast to the *T. b. rhodesiense* induced anemia in mice, the PCV of the non-infected control mice increased from 52.9±2.2 at day 0 to 54.9±1.1 at 14DPI, an increase of 3.8% ( Figure 5).

### Body weight changes

**Figure 6.**
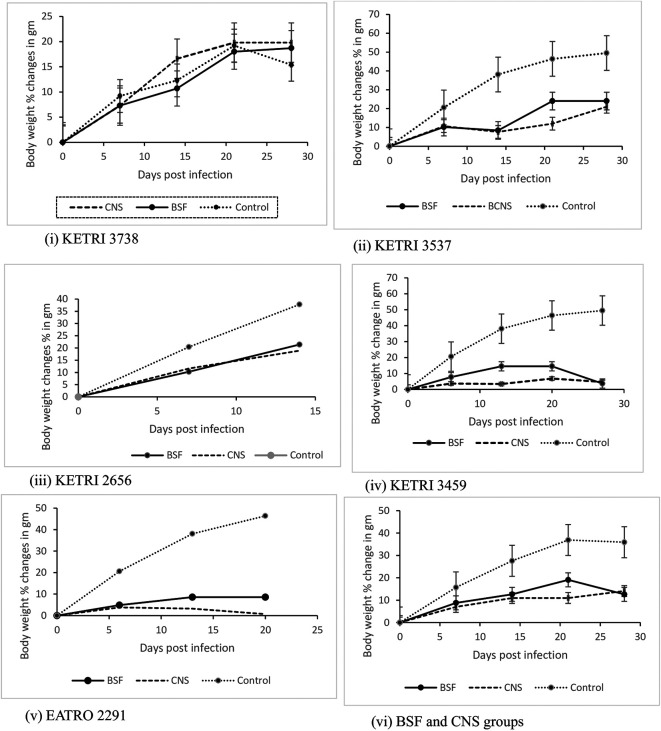
Graph showing the body weight changes in mice infected with BSF or CNS derived trypanosomes with days post infection. Abbreviations: BSF, blood stream forms; CNS, central nervous system. Data are mean ±SE of mice body weight changes (n=10).

The mean ±SE pre-infection body weight data was 27.6±1.8 and 27.1±1.8gm for BSF and CNS groups respectively and 21.7±2.2 for the non-infected control group. The body weight of the non-infected control group (n =5) increased by 28% from a baseline (day 0) value of 21.7±2.2g to 27.7±0.8 g at 14 days post infection; this increase was significant (p < 0.05). The infected mice groups also continued to gain weight during the duration of the experiment but the net weight gains exhibited by mice infected with isolates KETRI 2656, KETRI 3537, KETRI 3459 and EATRO 2291 were lower than the weight gains exhibited by control mice for each experiment ( Figure 6). For the mice groups infected with isolate KETRI 2291, the weight gains were minimal ( Figure 6). However, mice group infected with isolate KETRI 3738 gained weight equally well with the uninfected control mice ( Figure 6). Among the infected mice groups, KETRI 2656 BSF infected mice recorded the highest increase in body weight; their body weight increased by 21 % from a baseline (day 0) value of 24.3±1.1g to 29.5±0.6 g at 14 days post infection ( Figure 6(iii)).

*Survival Time*

All control mice survived up to the end of the experimental period of 30 days and their survival time data were therefore categorised as censored. The survival time in the infected mice varied between the isolates ( Table 3). In BSF infected mice, the shortest survival was recorded in mice infected with two isolates, EATRO 2291 and KETRI 2656; these mice groups had mean ±SE survival times of 13.5±2.7 and 18.7±1.4 days respectively. The two isolates also exhibited the shortest survival times in mice groups infected with CNS forms ( Table 3). Mice infected with

KETRI 3738 BSF or CNS trypanosomes survived to post 30 days of infection. The p values associated with Wilcoxon and Logrank tests of homogeneity for the BSF or CNS forms of individual isolate ranged between 0.1 to 0.5 and 0.1 to 0.3 respectively showing no significant difference between the groups both at early and longer survival times. Even when the survival time of all mice infected with BSF and CNS trypanosome forms were grouped together and compared, there was no statistically significant difference (p> 0.05).

### Gross and histopathology results

**Figure 7.**
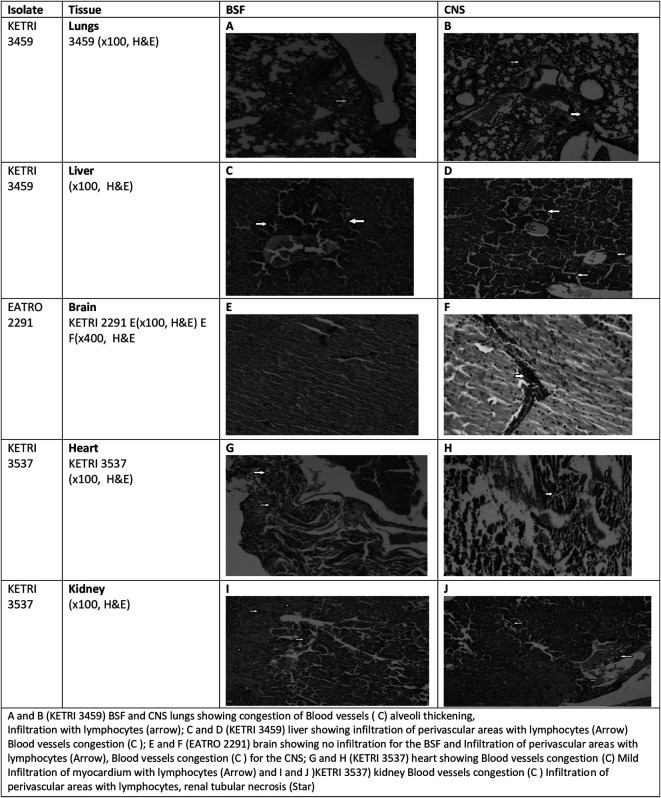
Showing the tissue pathology results observed in mice infected with BSF or CNS. Abbreviations: BSF, blood stream forms; CNS, central nervous system.

The gross pathology results revealed that hepatomegaly, splenomegaly and enlargement of lungs were common to all infected mice groups while cardiomegaly was only observed in mice groups infected with BSF forms of KETRI 3537 ( Table 5). At histopathology, it was observed that in general, tissue pathology in mice infected mice was characterized by congestion, hemorrhages, necrosis and infiltration with inflammatory cells including plasma cells, lymphocytes and macrophages around the blood vessels ( Figure 7).

**Table 5.** Organ weights of mice infected with BSF or CNS trypanosomes and non-infected control.

Isolate	Life b/wt	No of mice euthanized	Heart	L/kidney	R/kidney	Spleen	Liver	Brain	Lungs
KETRI 3738 BSF	32.5±2.7	4	0.2±0.01 (0.7)	0.28±0.02 (1.2)	0.28±0.03 (1.1)	1.16±0.2 (4.8)	2.7±0.2 (1.5)	0.39±0.04 (0.9)	0.32±0.06 (1.6)
**KETR 3738 CNS**	**34±1.1**	**4**	**0.18±0.02 (0.7)**	**0.25±0.01 (1.0)**	**0.26±0.01 (1.0)**	**0.18±0.09 (0.8)**	**3.4±0.3 (1.9)**	**0.43±0.01 (1.0)**	**0.31±0.02 (1.6)**
KETRI 3537 BSF **KETRI 3537CNS**	26.9±1.5	4	0.35±0.01 (1.3)	0.24±0.03 (1.0)	0.25±0.03 (1.0)	0.84±0.16 (3.5)	2.4±0.15 (1.4)	0.38±0.03 (0.9)	0.31±0.03 (1.6)
	**28.1±1.1**	**4**	**0.21±0.02 (0.8)**	**0.24±0.01 (1.0)**	**0.25±0.02 (1.0)**	**0.70±0.10 (2.9)**	**2.56±0.21 (1.5)**	**0.45±0.02 (1.1)**	**0.26±0.04 (1.3)**
KETRI 3459 BSF **KETRI 3459 CNS**	29.3±1.3	4	0.18±0.02 (0.6)	0.20±0.01 (0.8)	0.23±0.02 (0.9)	1.58±0.18 (6.6)	3.12±0.09 (1.8)	0.44±0.02 (1.0)	0.28±0.02 (1.4)
	**30.5±2.2**	**4**	**0.17±0.02 (0.6)**	**0.23±0.01 (1.0)**	**0.24±0.01 (0.96)**	**1.51±0.08 (6.3)**	**3.09±0.24 (1.8)**	**0.37±0.03 (0.9)**	**0.34±0.06 (1.7)**
EATRO 2291 BSF **EATRO 2291 CNS**	27.4±0.96	4	0.15±0.02 (0.6)	0.19±0.01 (0.8)	0.19±0.01 (0.8)	0.74±0.10 (3.0)	2.24±0.12 (1.3)	0.44±0.02 (1.0)	0.30±0.02 (1.5)
	**27.8±1.14**	**4**	**0.15±0.02 (0.6)**	**0.21±0.01 (0.9)**	**0.19±0.01 (0.8)**	**0.99±0.1 (4.1)**	**2.25±0.11 (1.3)**	**0.34±0.03 (0.8)**	**0.24±0.02 (1.2)**
Control	31.5±1.1	2	0.27±0.09	0.24±0.02	0.25±0.01	0.24±0.08	1.75±0.08	0.42±0.02	0.20±0.01

Numbers in parenthesis represent number of times the organ increased when compared with the control.

Abbreviations: b/wt, body weight, BSF, blood stream forms, CNS, central nervous system.

Hepatomegaly was common to all the infected mice groups, characterized by mean liver weights of infected mice being 1.3-1.8 times heavier than the mean liver weight of control mice Table 5. Histologically, the main lesions seen in liver tissue were infiltration with inflammatory cells around the centrilobular vein as well as in the parenchyma, dilated blood vessels, hemorrhage, bile duct distension and formation of new ductules; these lesions were present in all infected mice but were more pronounced in mice infected with CNS derived trypanosomes. In contrast, congestion, areas of necrosis and emphysema were more pronounced in BSF infected mice ( Figure 7C & D). Mean Spleen weights in the infected mice groups increased by a factor of 1.4 to 12.2 times of the mean weight of spleens in the negative control mice group Table 5. As with other tissues, the main lesions at histology were tissue infiltration with inflammatory cells, congestion and haemorrhage (data not shown).

Grossly the lungs were moderately enlarged by a factor of up to 1.6 times the weight of lungs recovered from the un-infected control mice. At histology, lesions in the lungs tissue were characterized by congestion of blood vessels, collapse and thickening of alveolar walls and infiltration of lung tissue with lymphocytes. These lesions were more pronounced in CNS infected mice as compared to BSF infected mice ( Figure 7A & B).

Cardiomegaly was observed only in mice that were infected with the BSF forms of KETRI 3537 and not in any other infected mice group Table 5. At histology, the main lesion observed was areas of necrosis in heart tissue and infiltration of inflammatory cells into the pericardium. Infiltration of inflammatory cells was more pronounced in BSF infected mice while areas of necrosis was more pronounced in CNS infected mice ( Figure 7G & H). Grossly, the brains were not enlarged Table 5. However, petechial hemorrhages were observed in the brains of mice infected with KETRI 3459 (data not shown). At histology, peri-vascular cuffing by inflammatory cells, vacuolation and areas of necrosis were the main lesions. These lesions were more pronounced in the CNS than in the BSF infected mice ( Figure 7E & F). Both left and right kidneys were grossly not enlarged Table 5. The kidney tissue showed more renal tissue infiltration with inflammatory cells in CNS than in BSF infected mice ( Figure 7I & J)

*Trypanosoma brucei rhodesiense*

A and B (KETRI 3459) BSF and CNS lungs showing congestion of Blood vessel (C) alveoli thickening, Infiltration with lymphocytes (arrow); C and D (KETRI 3459) liver showing infiltration of perivascular areas with lymphocytes (Arrow) Blood vessels congestion (C); E and F (EATRO 2291) brain showing no infiltration for the BSF and Infiltration of perivascular areas with lymphocytes (Arrow), Blood vessels congestion (C) for the CNS; G and H (KETRI 3537) heart showing Blood vessels congestion (C) Mild Infiltration of myocardium with lymphocytes (Arrow) and I and J)KETRI 3537) kidney Blood vessels congestion (C) Infiltration of perivascular areas with lymphocytes, renal tubular necrosis.

## Discussion

In this study, we recovered BSF and CNS trypanosome forms from 21-day old murine infections of five *T. b. rhodesiense* isolates and carried out a comparative morphology and pathogenicity study. Overall, our results showed differences in biomarkers of trypanosome pathogenicity in mice including survival time, pre-patent period, parasitaemia, PCV and reduced body weight gains in mice groups infected with BSF or CNS isolates. These results confirmed the existence of differential virulence among the isolates. Differential virulence and pathogenicity of trypanosomes has previously also been reported for other samples of *Tyrpanosoma brucei rhodesiense* isolates in mice, ^28^ *Trypanosoma brucei rhodesiense* isolates in vervet monkeys ^29^; *Trypanosoma brucei brucei* and *Trypanosoma congolense* strains in mice ^25^^,^^31^^–^^33^ and *T. evansi* isolates in mice. ^34^ In the present work, we have additionally shown that mice groups infected with CNS derived trypanosomes also exhibited differential virulence, indicating that this attribute is independent of the environment from which the trypanosomes are recovered.

The density of CNS trypanosome forms in brain supernatants was up to 1000 times lower than the density of BSF forms recovered from heart blood at the same time point, 21 days postinfection. This finding is in agreement with a previous report by ^35^ who found that, contrary to the high density of BSF trypanosomes, CNS trypanosome densities are very low. Recent progress in the understanding of the pathogenesis of trypanosome infections has shown that the parasites gain entry into the CNS compartment as early as 6-7 DPI, ^35^^,^^36^ via the blood-brain barrier (BBB) or via blood-CSF barrier (BCB). ^5^^,^^35^ The trypanosomes increase in numbers as the infection progresses such that by 21-28 days post infection, the CNS infection is well established as evidenced by remarkable increase in parasite numbers and tissue pathology. ^36^ In our study, the CNS trypanosomes were predominantly the monomorphic and proliferative long slender forms unlike the BSF which were pleomorphic as previously reported. ^38^ We however did not score the CNS parasitaemia at the termination of our experiment to determine the parasite density. Despite the generally low density of CNS trypanosomes, mice infected with isolate KETRI 2656 recorded a relatively high density of 1 × 10 ^6^ trypanosomes/mL ( Figure 4(iii), Table 2 which was 10 times the density of other CNS trypanosomes infections, providing evidence of inter-isolate differences in growth characteristics. High trypanosome numbers are frequently, but not always, associated with increased pathogenicity. ^28^^,^^39^

We monitored the development of anaemia which is one of the major trypanosome-induced pathologies and is a possible biomarker of parasite pathogenicity and virulence. The main characteristic of the murine trypanosome-induced anaemia was the rapidity of its onset in all the infected mice as shown by PCV declines by an average of 30% by 14 days post infection. This early phase of rapid PCV decline occurred concurrently with the first wave of parasitaemia in which parasites first appeared in blood at 4-5 days post infection and rose to peak levels by 8-9 days post infection. The rapid development of anemia in African tyrypnosomiasis has been postulated to be due to a mechanism involving enhancement of erythrophagocytosis by galectin 3 (Gal-3) and promotion of myeloid cell recruitment and iron retention within the mononuclear phagocyte system (MPS) which reduces iron reserves that are available for erythropoiesis and hemodilution. ^40^ The reduced availability of iron for erythropoiesis is likely to be responsible for previous research findings that the *T b rhodesiense* induced anaemia in animal models of human African trypanosomiasis of the microcytic hypochromic type. ^29^^,^^41^ Beyond 14 DPI, the PCV stabilized in mice infected by BSF or CNS derived trypanosomes despite the fact that parasitaemia remained high ( Figure 5) which is a characteristic of the chronic phase of anaemia. ^42^ Chronic phase anaemia is likely due to changes in cytokines such as IL10 that modulate tissue pathology. ^41^ The development and progression of anaemia during the acute and chronic phases of the experimental *T.b. rhodesiense* infections in mice disease was comparable in mice infected with either CNS and BSF forms ( Figure 5). Anaemia has been a widely documented pathology cases of HAT in humans ^43^ and various types of animal models. ^29^^,^^42^^,^^44^^,^^45^

An interesting observation in our study was that a majority of the mice continued to gain weight in spite of *T b rhodesiense* infection. However, the weight gains in infected mice were characteristically lower weight gains in un-infected control mice ( Figure 6) implying that the effect of trypanosome infections in this model is to reduce net weight gains in mice. The finding that mice continued to gain weight in spite of being infected with trypanosomes was in agreement with previous studies in which mice were infected with *T. congolense* or *T. brucei brucei* ^25^^,^^46^ and *T. evansi* trypanosomes. ^34^ In other studies, however, authors reported declines in the body weights of trypanosome-infected animals ^46^^,^^47^ suggesting the unreliability of gross body weight changes as a biomarker of parasite pathogenicity and virulence. Indeed our results showed an increase in the body weight of mice infected with isolate KETRI 2656 which based on its short survival time may be classified as virulent. ^31^ We attribute the increasing body weight of the mice to the fact that the mice had not yet attained their maximum adult weight at the start of the experiment, and partly also to trypanosome induced organomegaly Table 5.

Organomegaly affecting the spleen and the liver was a major finding in all the infected mice while enlargement of the lungs and heart were restricted to mice infected with specific isolates Table 5. In some of the infected mice, the enlargement of the spleen was up to 12 times the weight of spleens recovered from un-infected control mice ( Table 5). Occurrence of hepatosplenomegaly in infected mice in our study is consistent with previous observation in both experimental animals and humans ^32^^,^^48^ as hemolymphatic stage pathologies. ^49^^–^^52^ Splenomegaly was associated to acute and post-acute phase of *Trypanosoma lewisi* infections of laboratory rats ^53^ which according ^53^ may be attributed to the proliferation of Lymphocytes. In our current study, a common finding observed in all tissues at histological level was infiltration of the tissues with various types of inflammatory cells, indicating that uncontrolled or poorly controlled tissue inflammation is partly responsible for the organomegaly seen grossly. Uncontrolled inflammation has been cited to be a major cause of pathogenicity during chronic parasite infections. ^54^

## Conclusion

Our results confirmed the existence of differential pathogenicity between blood isolates of *Tbr* and further demonstrated the conservation of this difference among the CNS derived trypanosomes. We further identified KETRI 2656 as a suitable isolate for acute menigo- encephalitic studies. Despite the fact that cerebrospinal fluid is known to be hostile to trypanosomes, our study results only found differences in the morphology and parasite densities of BSF and CNS derived trypanosomes but no consistent differences in the pathogenicity of the two forms. Finally, our study has reinforced the fact that anaemia, parasite densities in blood and CNS, survival time and net weight gain, as opposed to total weight changes, are useful biomarkers of the pathogenicity of trypanosome infections in animal models.

## Data availability

### Underlying data

BioStudies: Parasitaemia, PCV and body weight changes in KETRI 3738 infected mice, S-BSST766.

BioStudies: Parasitaemia, PCV and body weight changes in KETRI 3537 infected mice, S-BSST768.

BioStudies: Paraitaemia, PCV and body weight changes in KETRI 2656 infected mice, S-BSST767.

BioStudies: Parasitaemia, PCV and body weight changes in KETRI 3459 infected mice, S-BSST769.

BioStudies: Parasitaemia, PCV and body weight changes in EATRO 2291 infected mice, S-BSST770.

BioStudies: KETRI 3738 Trypanosome morphological length, S-BSST771.

BioStudies: KETRI 3537 Trypanosomes morphological length, S-BSST772.

BioStudies: KETRI 2656Trypanosomes morphological length, S-BSST773.

BioStudies: Survival times in mice infected with Tbr KETRI 3738, S-BSST774.

BioStudies: Survival times in mice infected with Tbr KETRI 3537, S-BSST775.

BioStudies: Survival times in mice infected with Tbr KETRI 2656, S-BSST776.

BioStudies: Survival times in mice infected with Tbr KETRI 3459, S-BSST777.

BioStudies: Survival times in mice infected with Tbr EATRO 2291, S-BSST778.

BioStudies: Gross pathology in mice infected with Tbr KETRI 3738 BSF or CNS trypanosomes, S-BSST779.

BioStudies: Gross pathology in mice infected with Tbr KETRI 3537 BSF or CNS trypanosomes, S-BSST780.

BioStudies: Gross pathology in mice infected with Tbr KETRI 2656 BSF or CNS trypanosomes, S-BSST781.

BioStudies: Gross pathology in mice infected withTbr KETRI 3459 BSF or CNS trypanosomes, S-BSST782.

BioStudies: Gross pathology in mice infected with Tbr EATRO 2291 BSF or CNS trypanosomes, S-BSST783.

BioStudies: Figure 1: Molecular identification of the Tbr isolates, S-BSST784.

BioStudies: Figure 2: The morphology of BSF or CNS trypanosomes at 21days post infection, S-BSST785.

BioStudies: Figure 3: The pre-patent period in mice infected with BSF or CNS trypanosomes, S-BSST786.

BioStudies: Figure 4: Parasitaemia progression in mice infected with BSF or CNS derived trypanosomes, S-BSST787.

BioStudies: Figure 5: The PCV changes in mice infected with BSF or CNS trypanosomes with days post infection, S-BSST788.

BioStudies: Figure 6: Body weight changes in mice infected with BSF or CNS trypanosomes with days post infection, S-BSST789.

BioStudies: Figure 7: Tissue pathology in mice infected with BSF or CNS trypanosomes, S-BSST790.

### Reporting guidelines

BioStudies: ARRIVE guidelines checklist, S-BSST792.
