# Supplementary material for: The pathogenicity of blood stream and central nervous system forms of Trypanosoma brucei rhodesiense trypanosomes in laboratory mice: a comparative study
Source: F1000Res. 2023 Nov 15;11:260. Originally published 2022 Mar 2. [Version 2] doi: 10.12688/f1000research.75518.2 (PMC10755267; doi:10.12688/f1000research.75518.2)
Supplement: Peer review history

# Reviewer report: Reviewer response for version 2

**DOI:** 10.5256/f1000research.156734.r223177

**Published:** 2023-12-28

**Authors:** Chukwunonso Francis Obi

**Affiliations:** 1 University of Nigeria, Nsukka, Enugu, Nigeria

The authors have adequately addressed the concerns I previously mentioned, and I do not have any additional inquiries.

Is the work clearly and accurately presented and does it cite the current literature?

Yes

If applicable, is the statistical analysis and its interpretation appropriate?

Yes

Are all the source data underlying the results available to ensure full reproducibility?

Yes

Is the study design appropriate and is the work technically sound?

Yes

Are the conclusions drawn adequately supported by the results?

Yes

Are sufficient details of methods and analysis provided to allow replication by others?

Yes

Reviewer Expertise:

Epidemiology of Parasitic Diseases; Parasite Infection Dynamics; Chemotherapy of Parasitic Diseases; Parasitic Drug Resistance; Ethno-veterinary Medicine; Molecular Parasitology and Parasitic Immunology

I confirm that I have read this submission and believe that I have an appropriate level of expertise to confirm that it is of an acceptable scientific standard.

# Reviewer report: Reviewer response for version 2

**DOI:** 10.5256/f1000research.156734.r223176

**Published:** 2023-11-23

**Authors:** Benoit Stijlemans

**Affiliations:** 1 Myeloid Cell Immunology Laboratory, Centre for Inflammation Research, Vlaams Instituut voor Biotechnologie (VIB), Brussels, Belgium; 2 Laboratory of Cellular and Molecular Immunology, Vrije Universiteit Brussel (VUB), Brussels, Belgium

I appreciated the efforts the authors made to address my questions/concerns and thus have no further questions. It is a very nice work that therefore I believe it will be of importance for the trypanosomatid community.

Is the work clearly and accurately presented and does it cite the current literature?

Partly

If applicable, is the statistical analysis and its interpretation appropriate?

I cannot comment. A qualified statistician is required.

Are all the source data underlying the results available to ensure full reproducibility?

Yes

Is the study design appropriate and is the work technically sound?

Partly

Are the conclusions drawn adequately supported by the results?

Partly

Are sufficient details of methods and analysis provided to allow replication by others?

Yes

Reviewer Expertise:

NA

I confirm that I have read this submission and believe that I have an appropriate level of expertise to confirm that it is of an acceptable scientific standard.

# Reviewer report: Reviewer response for version 1

**DOI:** 10.5256/f1000research.79399.r176018

**Published:** 2023-08-04

**Authors:** Chukwunonso Francis Obi

**Affiliations:** 1 University of Nigeria, Nsukka, Enugu, Nigeria

The authors, Kariuki Ndungu et al. compared the pathogenicity of bloodstream forms (BSF) and central nervous system forms (CNS) of five isolates of *Trypanosoma brucei rhodesiense* in Swiss White mice. The BSF and CNS trypanosomes were recovered from the heart and brain respectively following infection of donor mice and euthanasia on day 21 post-infection. The BSF and CNS trypanosomes were thereafter infected into Swiss white mice and the parasitaemia, packed cell volume (PCV), body weight, survivorship, trypanosome length, gross, and histopathology were evaluated for comparison of the inter-isolate pathological differences. Differential pathogenicity/virulence was observed amongst the five isolates studies but concerning BSF and CNS trypanosomes, no definite differences were observed.

 This manuscript describes quite interesting research about the pathogenicity/virulence of BSF and CNS trypanosomes. The study is well-designed, structured, and contains quite a number of the parameters expected to be used for such a study. In some parts of the manuscript, minor formal/linguistic errors can be corrected.

 In my opinion, there are not many aspects of the manuscript that need to be improved.

- What is the infective dose (number of trypanosomes) used in this study? This should be stated categorically.
- Check Table 2. The BSF trypanosome density for KETRI 3738. Is the figure there correct? What does the numbers in brackets under the BSF/CNS represent?
- Why did the authors select only the KETRI 3738, KETRI 3537, and KETRI 2656 isolates for the trypanosome length determination? Why not on all the isolates?
- In the Gross and histopathology section, the authors stated that “ *…and 2/5 of the control mice group were sacrificed at extremis for gross and histopathological examination.*” Were the control groups of mice also at extremis during euthanasia? If yes, you should explain why. If no, please revise your sentence.
- In the Parasitaemia progression section (result), the authors wrote (1.0x109 /Ml) in 3 or more places. Please, replace Ml with mL.

Is the work clearly and accurately presented and does it cite the current literature?

Yes

If applicable, is the statistical analysis and its interpretation appropriate?

Yes

Are all the source data underlying the results available to ensure full reproducibility?

Yes

Is the study design appropriate and is the work technically sound?

Yes

Are the conclusions drawn adequately supported by the results?

Yes

Are sufficient details of methods and analysis provided to allow replication by others?

Yes

Reviewer Expertise:

Epidemiology of Parasitic Diseases; Parasite Infection Dynamics; Chemotherapy of Parasitic Diseases; Parasitic Drug Resistance; Ethno-veterinary Medicine; Molecular Parasitology and Parasitic Immunology

I confirm that I have read this submission and believe that I have an appropriate level of expertise to confirm that it is of an acceptable scientific standard, however I have significant reservations, as outlined above.

# Response

**Published:** 2023-10-26

**Authors:** Kariuki Ndungu

**Affiliations:** Kenya Agricultural and Livestock Research Organization, Kenya

Reviewer comments ( **Chukwunonso Francis Obi)**

 Comment

 What is the infective dose (number of trypanosomes) used in this study? This should be stated categorically.

 Response

 We did not have a uniform infective dose that we used in all the experimental animals. However, for each trypanosome isolate, the infective dose for experimental mice infected with CNS and BSF trypanosomes was equal. Brain trypanosomes are usually too scanty to be diluted. We therefore determined the density of CNS trypanosomes and  adjusted the density  blood stream form trypanosomes to match this; this procedure was repeated for each of the isolates as shown below which shows that the infective dose was specific to each trypanosome isolate.

 This is now well captured in the manuscript: margin lines 98-104

 The inoculum doses for BSF or CNS groups of mice were:

 KETRI 3738 1.5 x 10 ^5^

 KETRI 3537 1.0 x 10 ^5^

 KETRI 2656 7.0x10 ^6^

 KETRI 3459 4.0 x 10 ^5^

 EATRO 2291 1.0 x10 ^5^

 Margin lines 98-104

 Comment

 Check Table 2. The BSF trypanosome density for KETRI 3738. Is the figure there correct? What does the numbers in brackets under the BSF/CNS represent?

 Response

 Yes the figure is correct. It represents the number the BSF trypanosomes were diluted (567 for KETRI 3738) to equal the CNS density whereas the number in bracket represents the antilog of 567 ). For clarity, we have inserted in the manuscript the sentence” ***The numbers in the fourth column represents the dilution factor used to ensure that the density of CNS and BSF trypanosomes was equal. We have reealised that the number in brackets is not adding any value to the narrative and have therefore deleted it from the table. We thank the reviewer for pointing this out. *** Margin lines 98-104

 Comment

 Why did the authors select only the KETRI 3738, KETRI 3537, and KETRI 2656 isolates for the trypanosome length determination? Why not on all the isolates?

 Response

 We carried out trypanosome length determinations in 3 out of the five study isolates due to compare the morphological characteristics of CNS and BSF forms of trypanosomes. The comparison was primarily between the two forms of trypanosomes for each isolate, hence we think that the data presented is valid. However, we concur with the reviewer and would like to state the reasons for limiting this aspect of the study to three isolates were logistics and resource based, rather than scientific considerations.  For clarity, we have inserted in the manuscript the sentence ***” Out of the five isolates used in this study, we randomly picked and-measured the length……..”*** margin lines 132.

 Comment

 In the Gross and histopathology section, the authors stated that “ *…and 2/5 of the control mice group were sacrificed at extremis for gross and histopathological examination.*” Were the control groups of mice also at extremis during euthanasia? If yes, you should explain why. If no, please revise your sentence.

 Response

 We appreciate this observation by the reviewer. We confirm that the control mice were not at extremis. The sentence has now been revised. Margin lines 146-1417

 Comment

 In the Parasitaemia progression section (result), the authors wrote (1.0x109 /Ml) in 3 or more places. Please, replace Ml with mL.

 Response

 This is now corrected as suggested by the reviewer. Margin lines 220-222

# Reviewer report: Reviewer response for version 1

**DOI:** 10.5256/f1000research.79399.r153045

**Published:** 2022-11-04

**Authors:** Benoit Stijlemans

**Affiliations:** 1 Myeloid Cell Immunology Laboratory, Centre for Inflammation Research, Vlaams Instituut voor Biotechnologie (VIB), Brussels, Belgium; 2 Laboratory of Cellular and Molecular Immunology, Vrije Universiteit Brussel (VUB), Brussels, Belgium

The manuscript entitled “The pathogenicity of the bloodstream and central nervous system forms of *Trypanosoma brucei rhodesiense* trypanosomes in laboratory mice: a comparative study” by Ndungu K, Thuita J, Murilla G, Kagira J, Auma J, Mireji P, Ngae G, Okumu P *et al.* documents on the comparison of the inter-isolate pathological differences caused by bloodstream forms (BSF) and central nervous system (CNS) of five *Trypanosoma brucei rhodesiense* ( *Tbr*) isolated from Swiss white mice. To this end, pre-infections in mice were performed using the 5 different stabilates after which parasites were isolated from either the bloodstream or the brain, referred to as BSF (bloodstream forms) and CNS (central nervous system forms). Subsequently, these isolated BSF (being LS and SS) and CNS (being predominantly LS) parasites were injected into new mice and infection parameters (including parasitemia, packed cell volume (PCV), body weight, survivorship, trypanosome length, gross and histopathology characteristics) were investigated. Though the five isolates showed differences with respect to virulence/pathogenicity no real difference between BSF and CNS forms from the same isolates were detected.

 This is an interesting study but I feel there are some aspects that are not clear or completely overlooked and which would bring added value to the manuscript. I will address each of the questions point by point.

**Is the work clearly and accurately presented and does it cite the current literature?**

- References are correctly cited.
- In Figure 2 the authors show the morphology of BSF or CNS trypanosomes at 21 DPI examined from giemsa-stained smears at 10x100 magnification using the oil immersion objective. This figure misses the image of KETRI 2656 BSF and also the image of KETRI 3459 CNS. I also do not see the added value of the image (vix) which by the way should be referred to as (ix).
- In Figure 7, immunohistological images are shown for selected organs of selected isolates. It should be explained why only these are shown because one would expect to have an overview of all isolates

**Is the study design appropriate and is the work technically sound?**

- The idea to compare parasites isolated from the blood or the brain is an interesting question. However, I think that there are several controls missing.
  - After washing the brains extensively the organ is homogenized and parasites present within this suspension are counted. Next, the concentration of parasites is normalized (BSF=CNS) and subsequently injected into new mice for the real experiment. I think here, there is an important control missing; mice that receive blood and homogenized brains from naïve mice (in a volume equal to used for the injection of parasitized samples). 
  - In addition, when looking at Figure 2 it seems that there is a lot of debris present in the CNS samples. In addition, as the authors stated there is an influx of immune cells in the brain during infection. Hence, it could be that within this homogenized preparation there are different cytokines/chemokine present that could affect the onset of infection and the final results (Table 3, time of peak parasitemia between BSF and CNS as well as between isolates; Fig. 6: weight change KETRI3738 compared to all others, Table 5 organ weights KETRI3738 such as spleen BSF versus CNS). Therefore, it would be important to measure these in the samples used to inoculate the experimental mice. To rule out any effect of debris that will be taken up by the cells from the newly infected mice, it would therefore be important to also include a group of mice receiving only homogenized brains (see point above). 

**Are the conclusions drawn adequately supported by the results?**

- The observation that the KETRI3738 strain behaves differently from the other strains is not really explained (see differences in Fig. 6: weight change KETRI3738 compared to all others, Table 5 organ weights KETRI3738 such as spleen BSF versus CNS).
  - For instance, in Figure 6 all infected animals gain weight to a much lesser extent than the control groups. However, why would there be no difference in weight change between the control and the BSF and CNS infected mice in the KETRI3738 strain but for all others, the infected animals lose weight?
  - Also, why is there almost no increase in spleen weight in the KETRI3738 CNS-infected animals but there is an increase in spleen weight in the BSF-infected animals? Were the RBC and immune cell numbers determined for the spleen because both could account for the splenomegaly?

Is the work clearly and accurately presented and does it cite the current literature?

Partly

If applicable, is the statistical analysis and its interpretation appropriate?

I cannot comment. A qualified statistician is required.

Are all the source data underlying the results available to ensure full reproducibility?

Yes

Is the study design appropriate and is the work technically sound?

Partly

Are the conclusions drawn adequately supported by the results?

Partly

Are sufficient details of methods and analysis provided to allow replication by others?

Yes

Reviewer Expertise:

Immunoparasitology, cellular immunology, African trypanosomiasis, nanobody-technology

I confirm that I have read this submission and believe that I have an appropriate level of expertise to confirm that it is of an acceptable scientific standard, however I have significant reservations, as outlined above.

# Response

**Published:** 2023-10-26

**Authors:** Kariuki Ndungu

**Affiliations:** Kenya Agricultural and Livestock Research Organization, Kenya

Reviewers comments

 Reviewer 1 ( **Benoit Stijlemans)**

 Comment

 In Figure 2 the authors show the morphology of BSF or CNS trypanosomes at 21 DPI examined from giemsa-stained smears at 10x100 magnification using the oil immersion objective. This figure misses the image of KETRI 2656 BSF and also the image of KETRI 3459 CNS. I also do not see the added value of the image (vix) which by the way should be referred to as (ix).

 Response

 We concur with the reviewer that for the purpose of comparison, we have only presented complete data for three isotes: EATRO 2291, KETRI 3537, and KETRI 3738; we also concur that the images of KETRI 2656 BSF and 3459 CNS are missing. We have corrected this by indicating in our results that ***“However, morphological data on KETRI 2656 BSF and 3459 CNS was not available for comparison”*** margin lines 192-193. We have therefore limited our discussion on this aspect of the study to the three isolates for which complete data is provided.

 We have also deleted image (vix) as suggested by the reviewer.

 Comment

 In Figure 7, immunohistological images are shown for selected organs of selected isolates. It should be explained why only these are shown because one would expect to have an overview of all isolates

 Response

 We appreciate the concern of the reviewer on the issue of histological images.  In the question of gross and histopathology, our results and discussion highlights the observations/comparison of lesions caused by BSF and CNS trypanosomes using the best images available to us from the study as examples.

 We have as a result inserted in the manuscript the sentence “For the comparison of the gross pathology and histology, we used the best images that were available from our results as examples” margin lines 315-316 However; we felt that the presented images were a good representative of the entire histological observation.(This is not supported by the text we have written on Figure 7)

 Comment

 After washing the brains extensively the organ is homogenized and parasites present within this suspension are counted. Next, the concentration of parasites is normalized (BSF=CNS) and subsequently injected into new mice for the real experiment. I think here, there is an important control missing; mice that receive blood and homogenized brains from naïve mice (in a volume equal to used for the injection of parasitized samples). 

 Response

 We are in agreement with the reviewer’s observation on the missing of this control. As such, we have inserted this as a limitation in our discussion ***“We however did not include as a control mice injected with brain homogenates and blood from naïve mice***”. Margin lines 357-358.

 However, there are important considerations which raise doubt on the value of such controls especially taking into consideration the need to use only the minimum number of animals required by animal welfare guidelines: considering that the route of infection of the experimental mice was intraperitoneal, would injecting diluted blood or brain homogenates from naïve mice have a significant impact on the pathogenicity parameters monitored in this study? Our answer is no. The parameters we monitored are related to presence of trypanosomes (e.g  prepatent period, parasitaemia patterns, gross and histopathology). We think that the suggested controls may not  significantly affect these parameters

 Comment

 In addition, when looking at Figure 2 it seems that there is a lot of debris present in the CNS samples. In addition, as the authors stated there is an influx of immune cells in the brain during infection. Hence, it could be that within this homogenized preparation there are different cytokines/chemokine present that could affect the onset of infection and the final results (Table 3, time of peak parasitemia between BSF and CNS as well as between isolates; Fig. 6: weight change KETRI3738 compared to all others, Table 5 organ weights KETRI3738 such as spleen BSF versus CNS). Therefore, it would be important to measure these in the samples used to inoculate the experimental mice. To rule out any effect of debris that will be taken up by the cells from the newly infected mice, it would therefore be important to also include a group of mice receiving only homogenized brains (see point above). 

 Response

 We thank the reviewer for these observations and acknowledge he has raised an important point that would be interesting to investigate in a subsequent study. We would however like to point out the following:

 The presence of debris in the CNS samples is likely related to the method of preparation of the brain samples (homogenization), as opposed to being infection related.

 While it is well established that cytokines/chemokines produced during a trypanosome infection can modulate the progression of the infection, It is not clear if cytokines/chemokines injected as part of the trypanosome inoculum fluid would have the same effect. In our study, we did not consider this to a major factor and therefore did not include it in our study design.  We note that at present, a majority of similar studies also use trypanosome isolates prepared from infected body fluids   

 Comment

 The observation that the KETRI3738 strain behaves differently from the other strains is not really explained (see differences in Fig. 6: weight change KETRI3738 compared to all others, Table 5 organ weights KETRI3738 such as spleen BSF versus CNS).

 Response

 We concur with the reviewer’s observation on our failure to explain the different behavior of KETRI 3738. In our view, this explanation is captured in our discussion on differential virulence demonstrated by both the BSF or CNS trypanosomes. We have however included in our discussion ***“This is even more pronounced in mice infected with KETRI 3738 whose response to infection was comparatively different (Fig 6 & Table 5)”*** margin lines 362-363.

 Comment

 For instance, in Figure 6 all infected animals gain weight to a much lesser extent than the control groups. However, why would there be no difference in weight change between the control and the BSF and CNS infected mice in the KETRI3738 strain but for all others, the infected animals lose weight?

 Response

 It is true there was no difference in body weight change between mice infected with KETRI 3738 and the non-infected control mice. To explain this, we have included in our discussion the sentence” ***We specifically did not observe any difference between mice infected with KETRI 3738 and the non-infected control suggesting the infection with this isolate did not affect their food appetite. However this we cannot confirm since we did not include food and water uptake measurement in our study”*** margin lines 414-417

 Comment

 Also, why is there almost no increase in spleen weight in the KETRI3738 CNS-infected animals but there is an increase in spleen weight in the BSF-infected animals? Were the RBC and immune cell numbers determined for the spleen because both could account for the splenomegaly?

 Response

 We believe response to this concern by the reviewer is captured in our responses to the above comments.
